# State-of-the-Art Molecular Plant Sciences in Brazil

**DOI:** 10.3390/ijms24108909

**Published:** 2023-05-17

**Authors:** Pedro Augusto Braga dos Reis, Jurandir Vieira Magalhaes, Robert Neil Gerard Miller, Elizabeth Pacheco Batista Fontes

**Affiliations:** 1 Department of Biochemistry and Molecular Biology, Universidade Federal de Viçosa, Viçosa 36570-000, MG, Brazil; bbfontes@ufv.br; 2 National Institute of Science and Technology in Plant–Pest Interactions, Bioagro, Viçosa 36570-000, MG, Brazil; 3 Embrapa Maize and Sorghum, Sete Lagoas 35701-970, MG, Brazil; jurandir.magalhaes@embrapa.br; 4 Biological Science Institute, Universidade de Brasília, Brasília 70910-900, DF, Brazil; robertmiller@unb.br

Brazil has a crucial role in global food security and biodiversity, boasting one of the largest agricultural areas and two globally vital biomes, the Amazon and the Atlantic Forest. Over recent decades, the Brazilian plant scientific community has contributed significantly to ensuring sustainable food production while preserving the environment, which has positively impacted food safety worldwide. However, climate change is enhancing environmental constraints for sustainable agricultural production. Warmer temperatures, increasingly erratic precipitation patterns, and water scarcity are influencing crop physiology, growth, and development, and, ultimately, triggering plant stress. Therefore, the continuous adaptation of plants to new or transitory environments has become imperative. Hence, a significant portion of agricultural research in Brazil is now focused on increasing food production while mitigating losses caused by sub-optimal environmental conditions—either biotic or abiotic. An increased understanding of how plants cope with these challenges is an urgent prerequisite for a sustainable environment.

Cutting-edge omics approaches, along with molecular and cell biology advancements, have enabled considerable evolution in plant sciences worldwide, resulting in the development of new tools and the improved resolution of existing ones. Therefore, Brazilian molecular plant science has also experienced continuous progress across numerous fields.

This Editorial focuses on the comprehensive molecular plant science research conducted in Brazil and emphasizes its main challenges and future perspectives. Comprising both model and crop plants, the Special Issue entitled “State-of-the-Art Molecular Plant Sciences in Brazil” highlights examples of cutting-edge omics approaches associated with molecular biology that are aimed at understanding plant responses to various conditions. Brazilian research in plant sciences is transversal, spanning from basic plant science to applications in Brazilian agriculture. This Special Issue published in the *International Journal of Molecular Sciences* consists of eleven contributions, including seven original research articles and four reviews, addressing different questions regarding plant development, stress response, and the generation of novel tools for plant science studies.

Although Brazil is a global leader in soybean and sweet orange juice production, these crops are continually affected by both abiotic and biotic stresses. To address how these crops might overcome such stress conditions, Longhi et al. [1] and Abruzzi de Oliveira-Busatto et al. [2] investigated stress tolerance strategies, providing new perspectives that may be applied to plant breeding and genetic engineering. In their study, Longhi and colleagues expressed the antimicrobial peptide (AMP) *sarcotoxin IA* (*stx IA*) gene from *Sarcophaga peregrina* in Pera sweet orange via Agrobacterium-mediated transformation. Transgenic lines displayed a reduced susceptibility to Huanglonbing (HLB) disease, a highly aggressive citrus disease, without affecting the development or fruit quality. Their results suggest that the *stx IA* gene may be a candidate for HLB tolerance. Focusing on flooding stress, Abruzzi de Oliveira-Busatto and colleagues identified candidate genes involved in flooding tolerance in soybean. They identified genes that were differentially expressed under flooding stress and between soybean genotypes, and that were contrasting in relation to flooding tolerance. Based on RNA-seq data, the authors identified over one thousand SNPs, with twenty-two SNP loci selected for field validation. Five markers displayed statistical significance with grain yield, indicating their potential for use in marker-assisted selection for flooding tolerance in soybean.

In their study, Campos et al. [3] assessed the influence of Ca^2+^ concentrations on mechanical damage signaling and photosynthesis using *Solanum lycopersicum* Micro-Tom plants. Mechanical damage activates reactive oxygen species (ROS) signaling, promoting the expression of stress-responsive genes. The study showed that combining Ca^2+^ supplementation and mechanical damage promotes a decrease in RuBisCO activity, which may be a mechanism for overcoming stress conditions. Additionally, this study indicates that the absence of Ca^2+^ impairs plant survival following mechanical damage.

Various methodologies can be employed to identify and understand plant cellular mechanisms in response to biotic stress, as well as to develop distinct molecular strategies to reduce negative effects. The studies by Pinheiro et al. [4] and Ribeiro et al. [5] showed different methods to evaluate plant response to pathogens and limit their deleterious effects. The sterility and resulting susceptibility of bananas (*Musa* spp.) to different pathogens led Pinheiro and colleagues to investigate the effect of *Pseudocercospora musae* on gene expression in *Musa acuminata* subsp. *burmannicoides*, var. Calcutta 4, a resistant fertile wild diploid, with the characterization of genes involved in the initial immune response. With over 500 differentially expressed genes identified that likely relate to immune responses, these results contribute to the development of new approaches for enhancing resistance against *P. musae*, which is currently difficult to achieve through conventional breeding. Ribeiro et al. employed RNA interference-mediated gene silencing to generate cotton transgenic lines expressing double-stranded RNA (dsRNA) for three potential target genes, namely chitin synthase 2 (*AgCHS2*), vitellogenin (*AgVg*), and ecdysis-triggering hormone receptor (*AgETHr*), aimed at controlling the cotton boll weevil (CBW). Their rationale was based on the structure present in viroid genomes to protect the dsRNA from the processing enzyme complex, resulting in an efficient insect pest control approach.

Santos et al. [6] systematically reviewed the molecular biology involved in the interaction between plants and *Moniliophthora perniciosa*, the causal agent of witches’ broom disease, which is highly detrimental to cocoa crops. This study addressed several questions regarding the disease mechanism, the genes involved in fungal pathogenicity, and in plant responses to the fungus, as well as molecular markers associated with fungal resistance. The collected information highlighted the prevalence of studies conducted by Brazilian scientists, emphasizing the relevance of cocoa to Brazilian agriculture and the need to further understand the cocoa—*Moniliophthora perniciosa* interaction.

Genome-wide association studies (GWAS) are useful for identifying markers associated with specific traits. In a study by Ribeiro and colleagues [7], GWAS was used on different maize panels to identify candidate genes associated with phosphorus (P) acquisition and root morphology. Two identified SNPs tagging MAPKKK and AGC protein kinase genes displayed an association with root morphology, P deficiency, and seedling dry weight, implicating the respective kinase proteins in the modulation of root architecture and P deficiency responses.

Artificial intelligence (AI) and bioinformatic approaches have become essential in plant science development. Machine learning algorithms have been shown to be crucial tools for identifying proteins and their function based on specific features rather than sequence conservation. In this regard, Silva et al. [8] developed RLPredictiOme, a tool that accurately and precisely predicts receptor-like proteins (RLPs) in plant genomes. RLPs are transmembrane proteins that play a significant role in cell signaling mechanisms. The identification and characterization of RLPs are essential in understanding signal perception and transduction in plants, and their application in stress and developmental signaling pathways.

Barreto et al. [9] conducted a comprehensive review of plant mitochondria metabolism and its changes and adaptations in response to stress conditions. They highlighted the critical roles of alternative oxidases (AOX) and uncoupling proteins (UCPs) in bypassing oxidative phosphorylation. This mechanism is associated with retrograde signaling and affects chloroplast function. Both genes are induced under stress conditions, and their overexpression results in improved fitness under stress. The advancement of functional genomic approaches has led to a better understanding of the mitochondrial function and its structural components, uncovering new paths for plant adaptation to stress conditions.

Biological nitrogen fixation has emerged as an essential alternative to minimize the adverse side effects of chemical fertilizers. Thiebaut et al. [10] addressed the use of non-nodulating diazotrophic bacteria for plant cultivation, highlighting the relevance and pioneering role of Brazilian science in bioinoculant research. To characterize the molecular mechanisms involved in the interaction between plants and diazotrophic bacteria, cutting-edge omics approaches have been employed, allowing for the identification of target genes and pathways essential for these mutually beneficial interactions. This research emphasizes the multipurpose use of biofertilizers, showing their potential also for biocontrol and stress effect mitigation.

Oliveira et al. [11] conducted an extensive review of the effect of phosphorylation on heterotrimeric G signaling and its impact on signaling dynamics and specificity. They mapped the in vivo and in vitro previously identified phosphorylation sites and their conservation across the plant kingdom. As plant genomes typically encode one or a few canonical heterotrimeric G-complex genes, signaling activation and discrimination rely on different phosphorylation codes. These so-called phosphocodes might be responsible for G-protein plasticity, and their characterization may aid in developing novel strategies for plant stress tolerance.

Overall, this Special Issue highlights the recent scientific achievements in plant molecular biology in Brazil, covering various topics across distinct fields. These studies promote a better understanding of the interaction between plants and their environment, offering a range of innovative solutions for achieving higher yields while maintaining a sustainable environment.
